# Understanding the Response of Poly(ethylene glycol) diacrylate (PEGDA) Hydrogel Networks: A Statistical Mechanics-Based Framework

**DOI:** 10.1021/acs.macromol.3c02635

**Published:** 2024-07-17

**Authors:** Michal Levin, Yongkui Tang, Claus D. Eisenbach, Megan T. Valentine, Noy Cohen

**Affiliations:** † Department of Materials Science and Engineering, Technion - Israel Institute of Technology, Haifa 3200003, Israel; ‡ Department of Mechanical Engineering, University of California, Santa Barbara, California 93106, United States; § Materials Research Laboratory, University of California, Santa Barbara, California 93106, United States; ∥ Institut for Polymerchemie, University of Stuttgart, Stuttgart D-70569, Germany

## Abstract

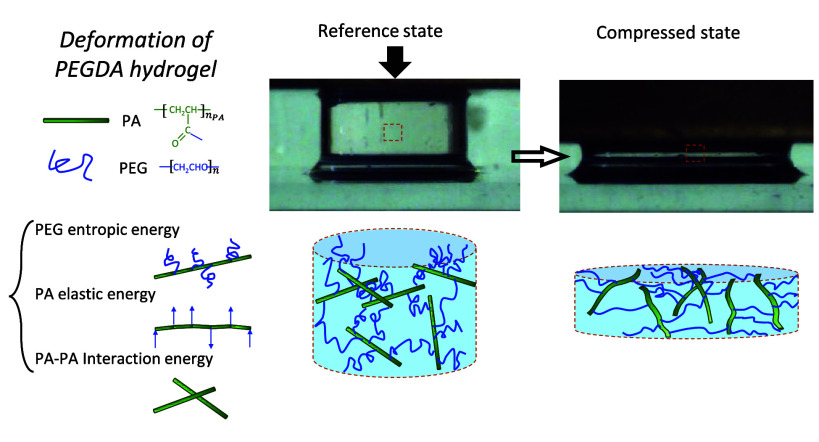

Thanks to many promising properties, including biocompatibility and the ability to experience large deformations, poly(ethylene glycol) diacrylate (PEGDA) hydrogels are excellent candidate materials for a wide range of applications. Interestingly, the polymerization of PEGDA leads to a network microstructure that is fundamentally different from that of the “classic” polymeric gels. Specifically, PEGDA hydrogels comprise PEG chains that are interconnected by multifunctional densely grafted rod-like polyacrylates (PAs), which serve as cross-linkers. In this work, we derive a microstructurally motivated model that captures the essential features which enable deformation in PEGDA hydrogels: (1) entropic elasticity of PEG chains, (2) deformation of PA rods, and (3) PA–PA interactions. Expressions for the energy-density functions and the stress associated with each of the three contributions are derived. The model demonstrates the microstructural evolution of the network during loading and reveals the role of key microscopic quantities. To validate the model, we fabricate and compress PEGDA hydrogel discs. The model is in excellent agreement with our experimental findings for a broad range of PEGDA compositions. Interestingly, we show that the response of PEGDA hydrogels with short PEG chains and long PA rods is governed by PA–PA interactions, whereas networks with longer PEG chains are dominated by entropy. To enable design, we employ the model to investigate the influence of key microstructural quantities, such as the length of the PEG and the PA chains, on the macroscopic properties and response. The findings from this work pave the way to the efficient design of PEGDA hydrogels with tunable properties and behaviors, which will enable the optimization of their performance in various applications.

## 1 Introduction

Hydrogels are soft materials with tunable mechanical and physical properties, which are governed by their chemical composition. The wide range of properties and versatility of hydrogels enables high innovation potential in material technology. The response of hydrogels under a mechanical loading is of particular interest for a wide range of applications, including tissue engineering,^1,2^ drug delivery,^3−5^ traction force microscopy,^6,7^ and microfluidics.^8,9^ In these systems, poly(ethylene glycol) (PEG) based hydrogels were used thanks to their stability under various conditions and biocompatibility.

**Figure 1:**
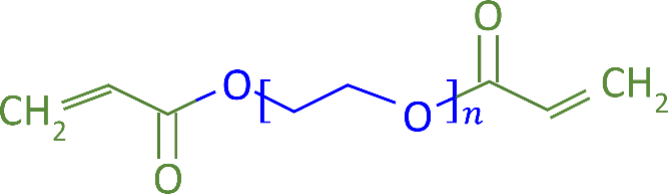
Chemical structure of PEGDA comprising a PEG chain (blue) and two acrylate (A, green) endings.

**Figure 2:**
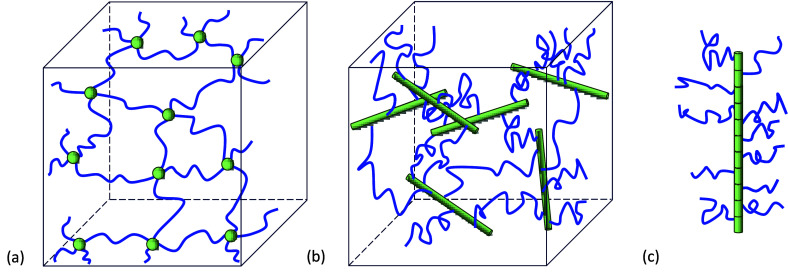
Three-dimensional schematic diagrams illustrating the differences between (a) “classical” and (b) bottlebrush hydrogel network structures. (c) Two-dimensional close-up schematic of an individual bottlebrush motif connecting a PEG chain to each PA repeat unit. PEG chains are shown in blue, and (a) PA junction points and (b, c) rod-like PA backbones in green. For clarity, (b) shows only a fraction of PEG cross-linking chains.

Such hydrogels were predominantly prepared by step-growth polymerization between multifunctional PEG with a multifunctional cross-linking agent,^3,4,10,11^ or by free radical polymerization of poly(ethylene glycol) diacrylate (PEGDA; Figure 1).^1,3,5−9^ The step-growth approach, e.g. by cross-linking bifunctional PEG with multifunctional cross-linkers of functionality *f* ≥ 3, results in relatively uniform PEG gel networks with point-like junctions. Figure 2a illustrates such an “ideal” hydrogel network formed using point-like tetra-functional junctions which are cross-linked by random-coil PEG chains. PEGDA photopolymerization, on the other hand, offers numerous advantages, including facile hydrogel formation as well as the ability to engineer complex multidimensional hydrogel structures.^12,13^ However, PEGDA forms significantly more complex networks, due to the creation of extended stiff polyacrylate (PA) chains that form the core of a complex bottlebrush structure.^14−16^Figure 2b illustrates a hydrogel of randomly oriented bottlebrush-like polymers with rod-like PA cores that are intertethered and connect random-coil PEG chains (Figure 2c).^17,18^

Critical to interpreting the structural and mechanical properties of PEGDA networks is understanding that each PEGDA macromonomer is capable of acting as both a monomer that enables the extension of PEG-based chains, as well as a cross-linking agent that connects two separate acrylate (A) cores. Notably, both acrylate groups on a single PEGDA macromonomer polymerize independently. Therefore, as the free radical photopolymerization of PEGDA proceeds, acrylate groups added to a radical PA chain end may originate from unreacted PEGDA macromonomers, or from PEGDA macromonomers in which one of the two acrylate end groups has already reacted and is fixed to another PA chain. This process results in a highly interconnected network topology. Moreover, adding acrylate to the growing PA rods necessarily involves the addition of PEG chains, which are grafted to the PA core. The steric requirements between PEG-grafted acrylate groups results in the formation of very stiff PA chains that occupy an extended rod-like conformation, while the softer PEG chains form random coils.^15,19−21^

Accordingly, the propagation reactions throughout the system result in a three-dimensional network structure consisting of rod-like PA chains, which are randomly oriented in space and serve as cross-links that interconnect entropic PEG chains. In this context, we point out that PEGDA-based hydrogels resemble a swollen molecularly reinforced composite (cf. refs (22, 23)) with stiff PA chains acting as reinforcer (similar to a fiber in a fiber-reinforced composite) in the swollen PEG matrix. The matrix-reinforcer coupling is perfect since each acrylate repeat unit is chemically linked to the PEG matrix. Topological defects such as loops and dangling ends carrying an unreacted acrylate group may be present in the network. However, it seems reasonable to assume such structural irregularities are negligible in view of the overall bottlebrush network structure that determines the mechanical properties and response of the highly cross-linked PEGDA hydrogel network.

From the literature data discussed above, it is clear that the structure of PEGDA-based hydrogels obtained by free radical polymerization is characterized by densely cross-linked bottlebrush polymers. In particular, this is concluded from SAXS and SANS data of a series of hydrogels made from PEGDA of different molecular weights,^18,24^ and estimates of the molecular weight of the polyacrylate chain, the key structural component of bottlebrush polymers (backbone), by selective ester cleavage of PEGDA-based networks.^25^

To the best of our knowledge, such complex bottlebrush networks have not been explored through theoretical modeling of PEGDA. Most available modeling approaches employ theories which either assume point-like junctions of functionality *f* = 3 to *f* = 4 or assume a star-like structure for *f* > 4.^17,24,26−31^ In the latter case, a structural model derived from experimental small angle neutron scattering data provided a systematic explanation of the linear elasticity and swelling of PEGDA hydrogels in which the PA backbone was assumed to be short relative to that of the PEG chains.^17^ However, this approach neglected considerable evidence that long bottlebrush structures are the key structural feature.^18,24,25^ In cases where the free radical photopolymerization of PEGDA results in a networks of bottlebrushes with multifunctional rod-like junction sites, the material structure is known to be quite distinct.^18,24−26,32^

The fundamental difference between the classical networks and the network formation and topology of PEGDA has far-reaching consequences on the mechanical properties and constitutive behavior of the hydrogel. The aim of this contribution is to better understand the structure–property relationships and the overall response of PEGDA hydrogels under mechanical loading. To this end, we derive a microstructurally motivated and energy-based model that captures the three essential features that enable deformation in PEGDA hydrogels: (1) the entropic contribution of the PEG chains, (2) the deformation of the PA rods, and (3) the mechanical PA–PA interactions. We demonstrate that in the limit of short PEG chains and long PA rods, the behavior is governed by PA–PA interactions, which we attribute to the mechanical restrictions due the high density grafting of PEG chains to the PA cores. In the case of long PEG chains and short PA rods, the hydrogel behaves entropically due to the deformation of the PEG chains. We also develop simple closed-form approximations for the expressions for the Young’s modulus and the stress. The model captures the key features of mechanical response and the microstructural evolution of the network during loading, and we show that it agrees with experimental findings for a broad range of PEGDA compositions.

## 2 Modeling the Response of PEGDA Networks

In developing our microstructurally motivated and energy-based model, we consider a PEGDA hydrogel network comprising *N*_*PEG*_ entropic PEG chains and *N*_*PA*_ stiff densely grafted PA chains per unit undeformed swollen volume. Each PA chain comprises *n*_*PA*_ acrylate repeat units and is connected to *n*_*PA*_ PEG chains. Accordingly, we can estimate *N*_*PA*_ ≈ 2*N*_*PEG*_/*n*_*PA*_.

To model the overall response of the PEGDA hydrogel, we denote the material points in a referential traction-free unswollen configuration by **X**. The network is placed in an aqueous bath and swells. In response to an external loading, the network deforms. The material points in the deformed state are denoted by **x** and we define the deformation gradient from the reference to the deformed state **F** = ∂**x**/∂**X**. The deformation can be written as **F** = **F**_*s*_**F**_*m*_, where **F**_*s*_ = *J*^1/3^**I**, where **I** is the identity matrix, is associated with the swelling of the network and **F**_*m*_ denotes the mechanical deformation due to the external loading. It is assumed that the mechanical loading is isochoric and accordingly det **F**_*m*_ = 1.

**Figure 3:**
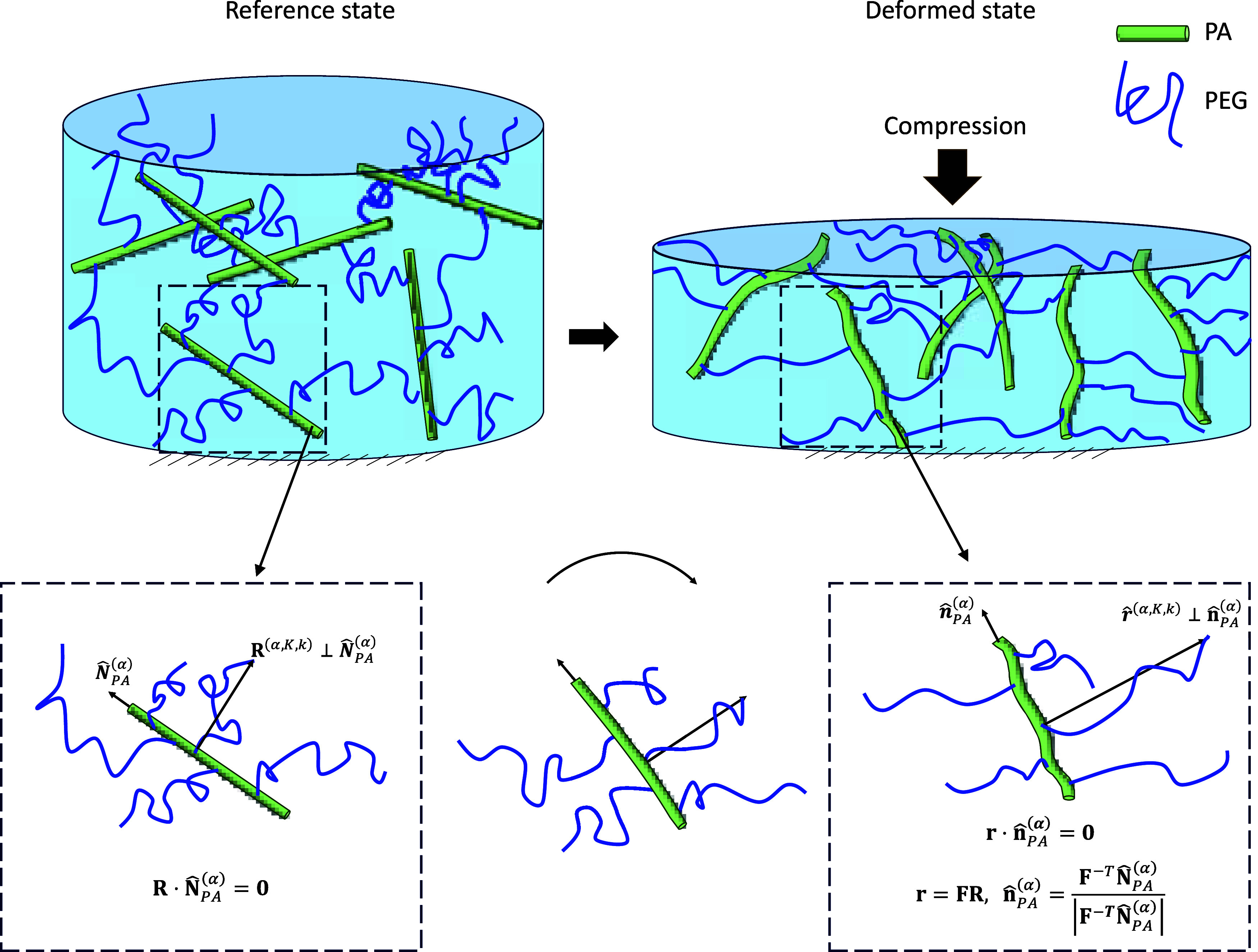
Reference and deformed state of PEGDA network. Under compression PEG chains oriented perpendicular to the loading direction entropically extend, thereby exerting forces that rotate and (slightly) bend the PA rods toward the loading direction.

The deformation of PEGDA networks is enabled by the entropic extension of the softer PEG chains, which transfer forces to the stiff PA chains, as illustrated in Figure 3. We assume that the total energy-density of the deformed gel can be viewed as the sum of three contributions:
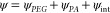
1where ψ_*PEG*_ is the entropic energy-density due to the deformation of the PEG chains, ψ_*PA*_ is the elastic energy-density resulting from the bending of the polyacrylate chains, and ψ_*int*_ is the energy-density due to the mechanical interactions between the PA chains.

Accordingly, the true stress is
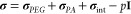
2where the σ_*PEG*_ = 1/*J*(∂ψ_*PEG*_/∂**F**)**F**^*T*^, σ_*PA*_ = 1/*J*(∂ψ_*PA*_/∂**F**)**F**^*T*^, and σ_*int*_ = 1/*J*(∂ψ_*int*_/∂**F**)**F**^*T*^ denote the stress associated with the PEG chains, the PA backbone, and the mechanical PA–PA interactions, respectively, and *p* is a work-less pressure-like term that stems from the incompressibility of the network and is determined from the boundary conditions.

It is also convenient to define the first Piola-Kirchhoff stress per unit swollen undeformed area:
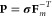
3

In the following, expressions for the three energy contributions described in eq 1 are derived. For convenience, Table 1 summarizes the model parameters that will be used.

**Table 1:** Summary of Model Parameters

Parameter	Definition
ψ_•_	Energy-density due to deformation of PEG chains (• = *PEG*), elastic bending of PA chains (• = *PA*), or PA–PA mechanical interactions (• = *int*)
**σ**_•_	Stress contribution due to the deformation of PEG chains (• = *PEG*), elastic bending of PA chains (• = *PA*), or PA–PA mechanical interactions (• = *int*)
**P**	Piola-Kirchhoff stress per unit undeformed swollen area
**F**	Deformation gradient
**F**_*s*_ = *J*^1/3^**I**	Deformation gradient associated with the swelling of the network
**F**_*m*_	Deformation gradient due to external mechanical loading
*N*_*PEG*_	Chain-density of PEG per unit undeformed swollen volume
*N*_*PA*_	Chain-density of PA rods per unit undeformed swollen volume
*n*	Number of freely jointed segments in a PEG chain
*n*_*PA*_	Number of acrylate repeat units in a PA rod
*L*_*PEG*_	Contour length of a PEG chain
*L*_*PA*_	Contour length of a PA rod
α	The maximum stiffness contribution due to PA–PA interactions in the limit *L*_*PEG*_/*L*_*PA*_ → 0
*J*	Ratio between swollen and dry volume of the PEGDA-based hydrogel network

### 2.1 Energy-Density of PEG Chains

The PEG chains are modeled as freely jointed chains (FJCs) with *n* freely jointed repeat units of length *l*. In the reference dry state, the end-to-end vector of the *i*-th chain is , where  and  are the end-to-end distance and the chain direction, respectively. In the swollen undeformed network, the chains stretch to accommodate water molecules such that the end-to-end distance increases , where *J* is the ratio between the swollen and the dry volume.^33−40^

Following common practice, we consider a network in which all chains assume the average referential end-to-end distance. By employing the affine deformations assumption, the deformed end-to-end vector of the *i*-th chain is
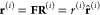
4where  and  are the deformed end-to-end distance and chain direction, respectively. We highlight that the deformed end-to-end distance depends on the orientation of the chain with respect to the loading direction.

The entropy of the *i*-th polymer chain is given by^35,41^
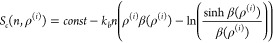
5where *k*_*b*_ is the Boltzmann constant, ρ^(*i*)^ = *r*^(*i*)^/*nl* is the ratio between the end-to-end distance and the contour length of the PEG chain, and β is determined from the Langevin function ρ = coth β – 1/β. A useful approximation that will be used in this work is β = ρ(3 – ρ^2^)/(1 – ρ^2^).^42^ The free energy of the *i*-th chain is ψ_*PEG*_^(*i*)^ = −*TS*_*c*_^(*i*)^, where *T* is the temperature, and accordingly the stress associated with the chain is
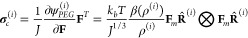
6

The overall energy of the PEG chains in the network is determined via
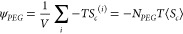
7where the summation is carried over all chains. Henceforth, the notation ⟨•⟩ = ∑_*i* = 1_^*N*^•^(*i*)^/*N* denotes the average of the quantity •. The stress that develops in the PEG chains is
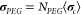
8where the stress associated with a chain **σ**_*c*_ is given in eq 6.

### 2.2 Energy-Density of PA Rods

As described in the introduction, the acrylate (A) groups in the PEGDA chains polymerize to form a PEG network with multifunctional cross-links in the form of densely grafted PA rods. Once the PEG chains stretch, the entropic forces work toward bending the PA rods, as illustrated in Figure 3. In the following, we model the elastic energy-density of the PA rods and their contribution to the overall macroscopic response. To this end, we begin by examining a single PA rod that is aligned along a given direction and derive an expression for its energy. Next, we sum over the energies of all PA rods along that direction. To determine the overall energy, an additional integration process over all PA direction is carried out. Following the chemistry of the PEGDA network, the PA chains are treated as stiff rods that undergo bending due to the entropic forces exerted by the PEG chains, which are grafted to them.

**Figure 4:**
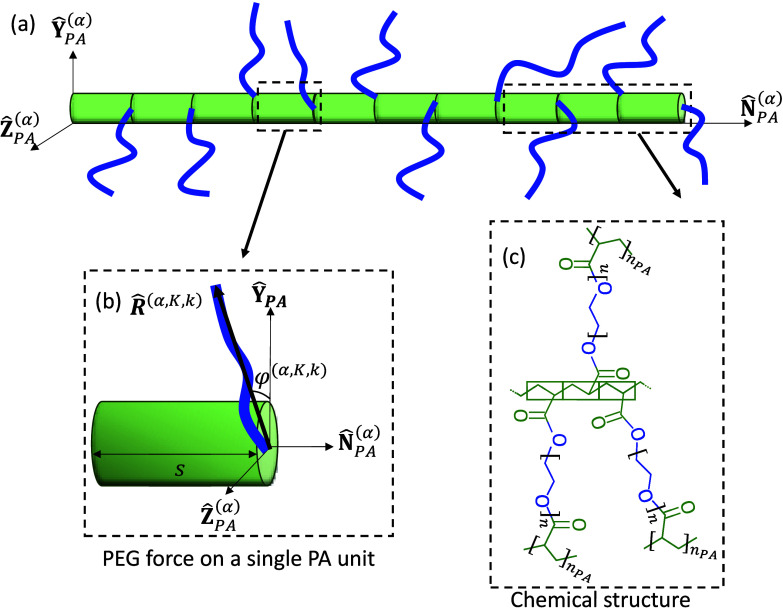
Representative part of the molecular structure of the PEGDA-based interconnected bottlebrush network: (a) A schematic of a PA rod pointing along the  direction that connects *n*_*PA*_ = 10 randomly oriented PEG chains. (b) A representative entropic force from the PEG chain along the PA rod . (c) Zoom in on the PA backbone, showing three acrylate repeat units with pendant PEG chains, each connected to adjacent bottlebrushes.

First, consider the *N*_*PA*_^(α)^ PA chains in the PEGDA network which are aligned along the direction  in the reference configuration. Here, the superscript α is used to denote a specific PA direction. It is convenient to define a local coordinate system , where  and  are unit vectors that span the plane perpendicular to the PA axis (see Figure 4). The average PA rod connects *n*_*PA*_ PEG chains that are equally spaced along the PA with a distance *l*_*A*_ = 0.25 nm (corresponding to the length of a repeat unit A) such that the overall length is *L*_*PA*_ = *n*_*PA*_*l*_*A*_.^15,43^ Next, we assume that (1) the PEG chains are perpendicularly connected to the PA such that their direction , (2) the entropic forces from the PEG chains are uniformly distributed along the PA chain, and (3) the effect of dangling ends is negligible.

Following assumption (1), the end-to-end vectors of the PEG chains along the PA backbone are found on the plane perpendicular to . To characterize this, consider one of the PAs aligned along . We mark this specific PA rod by an index *K*, where 1 ≤ *K* ≤ *N*_*PA*_^(α)^. This rod connects *n*_*PA*_ PEG chains, and we denote the end-to-end direction of the *k*-th PEG chain via
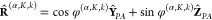
9where 0 ≤ φ < 2π is the angle between the end-to-end vector  and . Note that α, *K*, and 1 ≤ *k* ≤ *n*_*PA*_ denote the direction of the PA rod, the specific configuration of the interconnected PEG chains along the PA rod, and the location of the PEG chain along the PA rod, respectively (see Figure 4).

Upon the application of an external force, the PEG chains deform affinely (see eq 4) and rotate the PA rods. We denote the direction of the PA in the deformed configuration by  Assuming that the orientation of the PEG chains **r̂** with respect to the PA rods remains such that , we find that
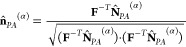
10

The entropic forces from the PEG chains result in the bending of the PA chain and are schematically illustrated in Figure 4. The force applied by the *k*-th PEG chain on the *K*-th PA rod aligned along  is
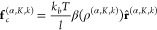
11

Accordingly, the moment at a distance *γl*_*A*_ ≤ ξ<(γ+1)*l*_*A*_, where 0 ≤ γ ≤ *n*_*PA*_ is an integer, can be written as
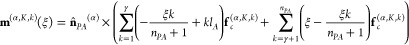
12The full derivation is given in section S1 of the Supporting Information. Next, the overall energy of the *K*-th PA rod can be computed via
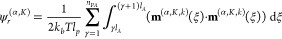
13Here, *l*_*p*_ = *EI*/*k*_*b*_*T* is the persistence length of a densely grafted PA rod, with *EI* denoting the bending stiffness. In addition, note that since the integration is along the length of the rod, the energy density ψ_*r*_^(α,*K*)^ does not contain the index *k*.

To determine the energy of all PA backbones aligned along the direction , we follow the assumption that the PEG chains are uniformly distributed and integrate
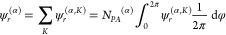
14The integral in the last equality denotes the average energy of a chain aligned along the direction  and the resulting energy ψ_*r*_^(α)^ does not contain the index *K*.

The average stress associated with the PA rods along the  direction can be determined from eq 14 via
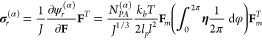
15where
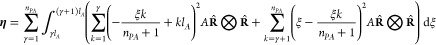
16and *A* = (2βdβ/d*r*)/(*ρn*), with dβ/d*r* = β^2^*sinh*^2^β/(*sinh*^2^β – β^2^).

Lastly, the energy-density of all PA chains is given by summing over all directions *N*_*PA*_^(α)^:
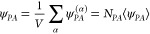
17Accordingly, the contribution of the PA rods to the overall stress is
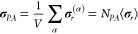
18

We point out that the stiffness of the PA rods stems from the collective effect of the densely grafted side-chains. Accordingly, two remarks are underscored. First, as opposed to the classical theories, the unique composition of PEGDA hydrogels results in a dependence between the entropic PEG chains and the PA rods. Specifically, adding monomers to the PA rod requires the addition of PEG chains. Accordingly, and as discussed in previous works,^44,45^ the classical definition of persistence length to bottlebrush polymer backbone is somewhat problematic and the contributions of the side-chains (or PEG in this case) must be taken into account. Due to steric hindrance, excluded volume, and intermolecular interactions, the addition of A units (i.e., an increase in *n*_*PA*_) may change the persistence length *l*_*p*_ of a PA rod. This agrees with interesting discussions in recently published works in the context of bottlebrush polymers in dilute and semidilute concentrations.^46−48^ Second, in networks with short PA rods that interconnect only a small number of PEG chains, the bending energy stored in the PA is negligible in comparison with the entropic energy of the PEG chains. Therefore, in such networks the contribution of the PA rods can be neglected.

### 2.3 Interaction Energy-Density of PA Backbone

**Figure 5:**
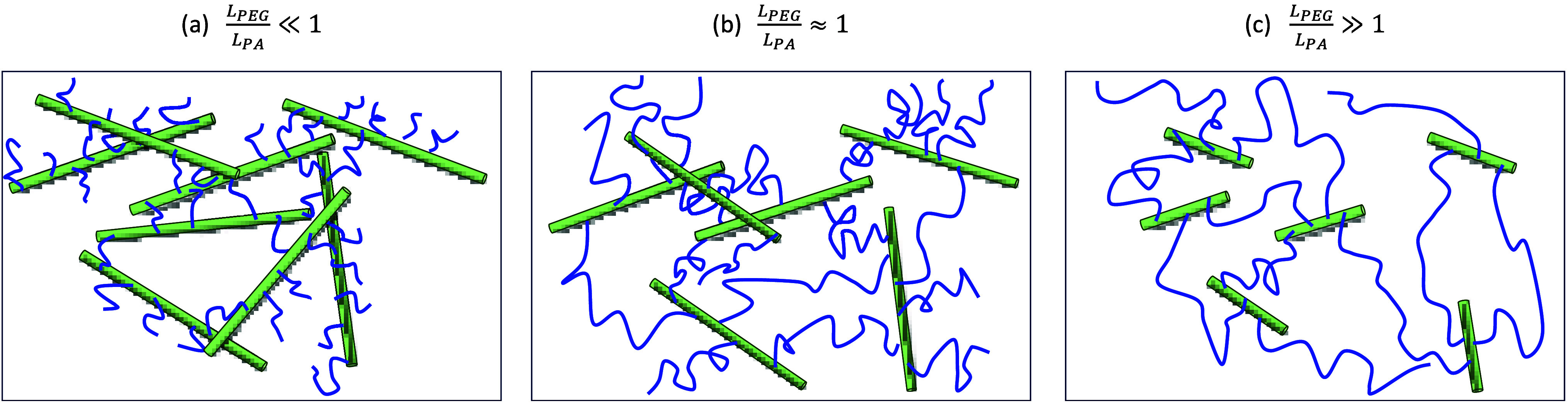
Illustration of a PEGDA network with (a) *L*_*PEG*_/*L*_*PA*_ ≪ 1, (b) *L*_*PEG*_/*L*_*PA*_ ≈ 1, and (c) *L*_*PEG*_/*L*_*PA*_ ≫ 1. In the case of *L*_*PEG*_/*L*_*PA*_ ≪ 1, PA rods are more likely to mechanically interact than *L*_*PEG*_/*L*_*PA*_ ≫ 1.

As opposed to the classical networks, during the formation of PEGDA hydrogels, A groups connect to form a network of PA rods that interconnect to one another through PEG chains. As a result, the PA rods can interact mechanically, thereby significantly changing the underlying mechanics of the system. In this context, we emphasize that the PA rods are not likely to physically come into contact. Rather, mechanical interactions include steric restrictions of the bottlebrush polymers due to their shape anisotropy. These restrictions are governed by two main factors: (1) the rotational freedom of the PA rods due to their hydrodynamic volume and (2) the ratio between the lengths of the PA rods and the PEG chains. The former is caused by the entropic forces from the PEG chains which act on the PA rods. To account for the latter, we examine the ratio *L*_*PEG*_/*L*_*PA*_ to account for these interactions (see Figure 5).

Recall that *L*_*PEG*_ = *nl* and *L*_*PA*_ = *n*_*PA*_*l*_*A*_ are the contour lengths of the PEG chain and the PA backbone, respectively. In the case of *L*_*PEG*_/*L*_*PA*_ ≪ 1 the PEG chains are significantly shorter than the PA backbone. Due to the stochastic nature of network formation, a PA rod can be interconnected to many other PA rods through mediating PEG chains, thereby increasing the density of PA rods per unit volume. As a result, mechanical interactions between PA rods is highly probable and is expected to affect the overall response. Since the PA rods are stiffer than the PEG chains, such interactions result in the stiffening of the network. If *L*_*PEG*_/*L*_*PA*_ ≈ 1, the likelihood of mechanical interactions is lower, but still exists and results in a slight stiffening. In networks with long PEG chains such that *L*_*PEG*_/*L*_*PA*_ ≫ 1, the PA backbones are far from each other and any PA–PA interactions can be neglected.

To model this effect, we propose the simple first-order interaction energy
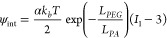
19where *I*_1_=Tr(**F**^*T*^**F**) is the first invariant and α denotes the maximum stiffness contribution of the interactions in the limit *L*_*PEG*_/*L*_*PA*_ → 0 (or in the case in which the PA rods are very close to each other). It is emphasized that in the limit *L*_*PEG*_/*L*_*PA*_ → *∞*, the PA backbones do not interact and the energetic contribution ψ_*int*_ → 0. The interactions between the PA backbones depends on their density, and therefore it is reasonable to assume that α ∼ *N*_*PA*_.

The stress due to these intermolecular interactions is determined from eq 19 via
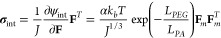
20

### 2.4 Integration from the Local Chains to the Network Level

To integrate from the chain to the network level, we employ the microsphere technique. This numerical method enables one to determine an average stress contribution by integrating over the stress associated with the individual chains. This technique was first introduced in ref (49) and later implemented to capture the response of polymeric networks.^37,50−54^

Consider a network with chains that are initially uniformly distributed and randomly oriented. The end-to-end vector directions  can be represented as unit vectors that begin at the center and end at the surface of a unit sphere. The directional averaging of a quantity • over the surface of the unit sphere can be approximated via the discrete summation
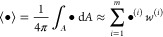
21where the index *i* = 1, 2, ..., *m* refers to the value of •^(*i*)^ along the *i*-th direction and *w*^(*i*)^ are non-negative weight functions constrained by ∑_*i* = 1_^*m*^*w*^(*i*)^ = 1. It is important to note that due to the uniform distribution and random orientations or the PEG chains, the averages  and .

In this work, we follow the findings of ref (49), which demonstrated that a specific choice of 42 integration directions provides sufficient accuracy in the computation of averages. The integration directions and the corresponding weights are given in Table 1 of ref (49).

### 2.5 Closed-Form Approximation for the Stress in PEGDA Hydrogels

The full model involves numerical integrations and the use of the microsphere approach, which leads to long computational times and makes it harder to explicitly determine pertinent quantities such as stiffness and overall response. To gain deeper insight into the overall microstructural evolution, simpler and efficient closed-form solutions are required. In the following, we derive a closed-form approximation for the stress that develops in PEGDA hydrogels. To this end, we consider a network with PEG chains and PA rods that are initially randomly oriented and uniformly distributed.

The stress that develops in the PEG chains can be approximated by considering the ratio ρ = *r*/*nl* ≪ 1. Accordingly, the PEG chains can be treated as Gaussian chains and the Langevin function can be approximated via a first order Taylor series as β ≈ 3ρ. The resulting stress associated with the deformation of the PEG chains is
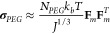
22

Next, we approximate the stress that develops in the PA rods. To this end, we take a second order Taylor series in ρ around zero in eq 15, substitute the resulting expression into eq 18, and compute the averages (see section S2 in the Supporting Information). This approximation yields
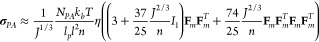
23where
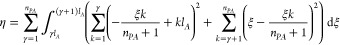
24and *I*_1_ = Tr(**F**_*m*_**F**_*m*_^*T*^) is the first invariant.

The interaction stress given in eq 20 is a first order approximation, and accordingly the total stress is estimated by substituting eqs 22, 23, and 20 into eq 2. The first Piola-Kirchhoff stress is determined from eq 3.

## 3 Comparison to Experiments

### 3.1 Experimental Protocol

Hydrogel disks with high geometric and compositional uniformity were fabricated under oxygen-free argon atmosphere by using a 3 mm thick PDMS plate with cylindrical bores of 6 mm in diameter sealed by a light-transparent coverslip at both sides. After filling the bottom-sealed cylindrical cavities with deionized aqueous solution of PEGDA containing lithium phenyl (2,4,6-trimethylbenzoyl) phosphinate (LAP) as photoinitiator (4.95 mM) and sealing the upper opening, gel samples were obtained by free radical photopolymerization (5 min irradiation at 370 nm with 4 mW/cm^2^ from both sides). In the gel syntheses, PEGDA concentrations *c* in the preparation state were above the critical overlap concentration c* of the corresponding PEG chain (recall that c* is the inverse of the intrinsic viscosity of PEG^55^). By setting *c* > c*, we avoid significant inhomogeneities in the PEGDA hydrogel network.^24,56,57^ Depending on the number-averaged molecular weight *M*_*n*_ of PEGDA, determined by gel permeation chromatograhpy (GPC with PEG calibration) to be *M*_*n*_ = 1.07 kDa, *M*_*n*_ = 2.03 kDa, and *M*_*n*_ = 5.85 kDa, the concentration *c* varied between ∼8–26%wt. For each PEGDA of given *M*_*n*_, three concentrations *c* were used.

To determine the volumetric deformation *J* (or the water content in the equilibrium swollen state of the PEGDA hydrogel discs), we weighed the hydrogel discs in the fully swollen and the dehydrated states. The volumetric deformation *J*, defined as the ratio between the swollen and the dry volumes of the gel, can be computed via
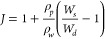
25where ρ_*p*_ ≈ 1.18 g/cm^3^ and ρ_*w*_ ≈ 1 g/cm^3^ are the densities of PEGDA and water, respectively, and *W*_*s*_ and *W*_*d*_ are the weights of the fully swollen gel and the dry polymer, respectively. We point out that different works report densities in the range of ∼1.07–1.18 g/cm^3^ of PEGDA.^26,27,29,58^

**Figure 6:**
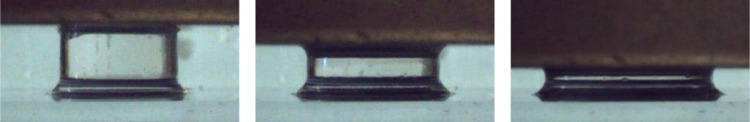
Side view of a uniaxial compression test on a cylindrical sample with molecular weight *M*_*n*_ = 2.03 kDa and concentration *c* = 18.4%wt/wt at the beginning (note that the uncompressed disk is 6 mm in diameter), the middle, and toward the end of the experiment.

Uniaxial compression tests were carried out on the fully swollen samples using a texture analyzer (TA.XT Plus Connect, Stable Micro Systems) with a 50 mm-diameter metal plate fixture attached to a vertically movable load cell. Before each compression test, to prevent the hydrogel from sticking to fixtures, a silicone oil coating was applied to the top and the bottom surfaces of the hydrogel as well as the metal fixtures of the testing device. Compression tests were executed at a rate of 0.01 mm/s up to rupture. Figure 6 shows images from the beginning, the middle, and toward the end of a uniaxial compression test on a cylindrical sample with molecular weight *M*_*n*_ = 2.03 kDa and concentration *c* = 18.4%wt/wt. A video of the experiment is given in movie SV1 with a description in section S3 of the Supporting Information. To ensure reproducibility, 3 specimens of each molecular weight and concentration were tested. The results are highly reproducible.

### 3.2 Model Fit

#### 3.2.1 Approximation - Uniaxial Tension/Compression

In the following we define a global deformation gradient  and examine the case of uniaxial compression. Accordingly, the deformation gradient due to a mechanical loading can be written as
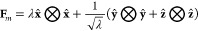
26

To determine the Piola-Kirchhoff stress, we employ the boundary conditions  to obtain the pressure term *p*. In the limit of small to moderate deformation, we employ the approximations in eqs 22 and 23 to obtain
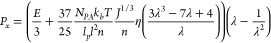
27where
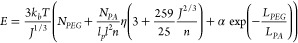
28is the Young’s modulus of the PEGDA network. Here, the quantity η is given in eq 24. In the case of *L*_*PA*_/*L*_*PEG*_ ≪ 1, it can be shown that the second and the third terms in eq 28 are negligible in comparison to the first and accordingly the classical expression for the Young’s modulus *E* ≈ 3*N*_*PEG*_*k*_*b*_*T*/*J*^1/3^ is recovered.^34,35^

#### 3.2.2 Model Fit

To validate the model, we compare its predictions to experimental findings. To this end, we consider three molecular weights *M*_*n*_ of PEGDA and three concentrations *c* for each molecular weight. The temperature is *T* = 300 K and we assume an interaction stiffness α = *N*_*PA*_. In addition, the acrylate length *l*_*A*_, the persistence length *l*_*p*_ of the densely grafted PA rod, and the length of a freely jointed repeat unit in the PEG chain *l* are approximated according to existing experimental data. Specifically, *l*_*A*_ = 0.25 nm,^15,43^*l*_*p*_ = 17.5 nm,^20,59,60^ and *l* = 0.3 nm.^61^ The number of repeat units in the average PEG chain is estimated via *n* ∼ *M*_*n*_/*M*_*EG*_, where *M*_*n*_ and *M*_*EG*_ ≈ 44g/mol are the molecular weights of the PEG in PEGDA (i.e., PEGDA without the end groups (A)), and an EG monomer repeat unit, respectively. The contour length of a PA rod *L*_*PA*_ and the chain-density of PEG *N*_*PEG*_ were determined based on a fit to the experimental data. We comment that our experiments show that the dry PEGDA network is brittle, suggesting that the response is not entropic. For convenience, the parameters are listed in Table 2.

**Table 2:** Properties of PEGDA Hydrogel Networks^a^

*M*_*n*_^#^ (kDa)	*n*^#^	*L*_*PEG*_^#^ (nm)	*c*^#^*%wt*/*wt*	*J*^#^	*n*_*PA*_	*L*_*PA*_ (nm)	*k*_*b*_*TN*_*PEG*_ (kPa)	ρ_0_^#^	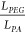
1.07	24	7	17.9	5.5	90	22.5	20	0.36	0.32
			21.8	4.8	95	23.75	29	0.34	0.3
			25.7	4.2	100	25	37	0.33	0.29
2.03	46	13	15.8	8.9	65	16.25	42	0.3	0.85
			18.4	7.6	70	17.5	53	0.29	0.79
			21.0	6.9	75	18.75	60	0.28	0.74
5.85	133	37	8.0	35.7	30	7.5	30	0.29	2.46
			11.9	22.2	47	11.75	45	0.24	2.28
			15.9	16.9	53	13.25	65	0.22	2.13

a Quantities marked with a ^#^ are measured directly or determined from the chemistry. *M*_*n*_: molecular weight of PEGDA, *n*: number of PEG repeat units in PEGDA; *L*_*PEG*_: estimated contour length of PEG chain based on a repeat unit length *l* ≈ 0.3 nm; *c*: experimental measurement of PEGDA concentration by weight; *J*: experimentally measured volumetric deformation; *n*_*PA*_: number of A repeat units in PA; *L*_*PA*_: contour length of a PA rod; *N*_*PEG*_: chain-density of PEG; : ratio between initial end-to-end distance and contour length of PEG chain.

**Figure 7:**
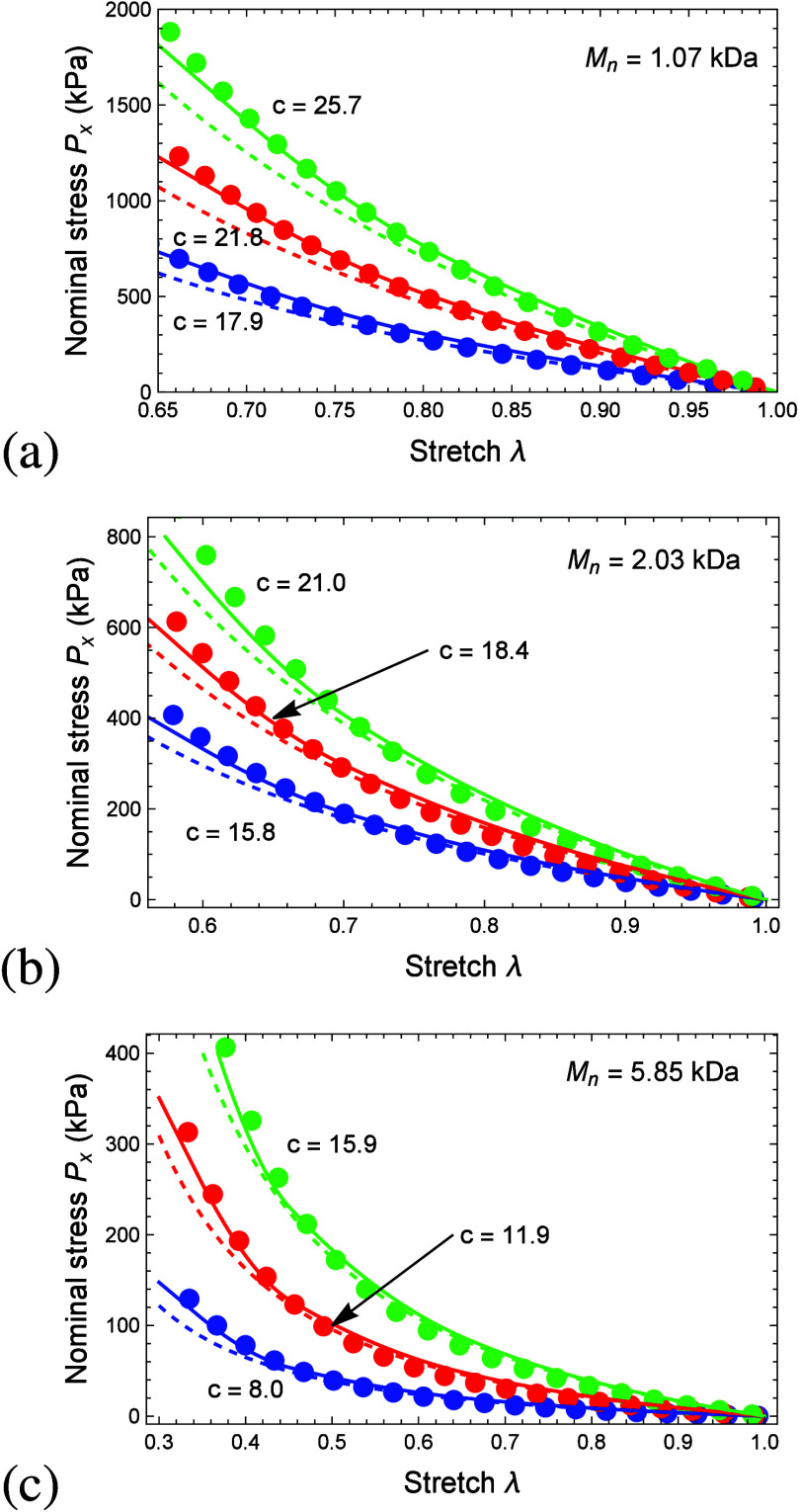
Comparison of the model to the experimental findings for (a) *M*_*n*_ = 1.07 kDa, (b) *M*_*n*_ = 2.03 kDa, and (c) *M*_*n*_ = 5.85 kDa. The circular markers denote the experimental findings, the continuous curves the full model, and the dashed curve the approximation in eq 27. The errors are smaller than the marker size.

The fit of the model and the approximation in eq 27 to the experimental findings for PEGDA hydrogel networks with the molecular weights *M*_*n*_ = 1.07 kDa, *M*_*n*_ = 2.03 kDa, and *M*_*n*_ = 5.85 kDa are shown in Figure 7a–c, respectively. The different concentrations are denoted in the plots. Here, the circle marks denote the experimental findings, the continuous curves the full model, and the dashed curve the closed-form approximation in eq 27. We find that the approximation provides a great fit for small to moderate stretches, with a maximum error of <17%.

We find that as we increase the length of the PEG chains (i.e., increase *n*), the predicted length of the PA backbone decreases, resulting in a lower *n*_*PA*_. We believe this to be a consequence of steric hindrance and intermolecular interactions between the PEG chains during the gel synthesis. In addition, the model predicts that increasing the concentration of PEGDA while maintaining a constant molecular weight *M*_*n*_ results in longer PA rods with the same PEG lengths (i.e., same *n* with larger *n*_*PA*_). Our experimental measurements show that this is also accompanied by a reduction in the volumetric deformation *J*. Such trends are to be expected from the kinetics of PEGDA hydrogel network formation.^23,62^

Another interesting finding is that the term *k*_*b*_*TN*_*PEG*_ increases with concentration *c* for a given molecular weight *M*_*n*_. However, comparison of this value under different molecular weights does not reveal a specific trend. These fits stem from the choice of α = *N*_*PA*_, which is set for all molecular weight (*M*_*n*_) values. Specifically, in the case of high molecular weights, such as *M*_*n*_ = 5.85, the overall behavior is governed by the entropic contribution of the PEG chains. In low molecular weights (i.e., *M*_*n*_ = 1.07 kDa), the stiffness may stem from two main sources: the bending energy and the mechanical interactions between the PA rods. The former is due to the high value of η, which results from the length of the rods. Given the proposed interaction energy and interaction stress (eqs 19 and 20) and the assumption that α = *N*_*PA*_, which denotes the maximum stiffness contribution of the mechanical PA–PA interactions in the limit *L*_*PEG*_/*L*_*PA*_ → 0, we find that the interactions between the PA rods are negligible in the examined PEGDA hydrogels. Such interactions are expected to be significantly more dominant as *L*_*PEG*_/*L*_*PA*_ → 0, and further investigations into the influence of the molecular weight *M*_*n*_ and the network composition on the parameter α and the interaction stress is required.

To emphasize the influence of the different stress components, we plot the contribution of the stress components *P*_*PEG*_, *P*_*PA*_, and *P*_*int*_ due to the deformation of the PEG network, the bending of the PA rods, and the PA–PA interactions, respectively, as a function of the stretch λ for hydrogel networks in Section S4, Figure S3 of the Supporting Information for three representative PEGDA networks. We show that in the case of *M*_*n*_ = 2.03 kDa and *c* = 15.8*%wt*/*wt*, the PEG chains are shorter than the PA rods and accordingly the stress component *P*_*PA*_ associated with the bending of the PA rods governs the response of the network. In networks characterized by *M*_*n*_ = 5.85 kDa and the concentrations *c* = 8.0*%wt*/*wt* and *c* = 15.9*%wt*/*wt* the chains are significantly longer than the PA rods. However, the water content in the two PEGDA networks is significantly different, resulting in variations in the extensions of the chains. Chains that experience larger stretches significantly stiffen the overall network.^35,37^ In all cases the interaction energy is negligible, and is expected to be more dominant as the ratio *L*_*PEG*_/*L*_*PA*_ → 0. However, we emphasize that a more rigorous experimental investigation into the interaction energy is required.

To further illustrate the influence of the bending of the stiff PA rods, we compare the model to the classical solutions from rubber elasticity of gels which consider point-like junctions^33,36,37^ in Section S5 of the Supporting Information. It is shown that while all models capture the right trend, neglecting the contribution of the PA rods results in a softer network. Therefore, it is essential to account for the contribution of the PA rods in the analysis of PEGDA networks.

**Figure 8:**
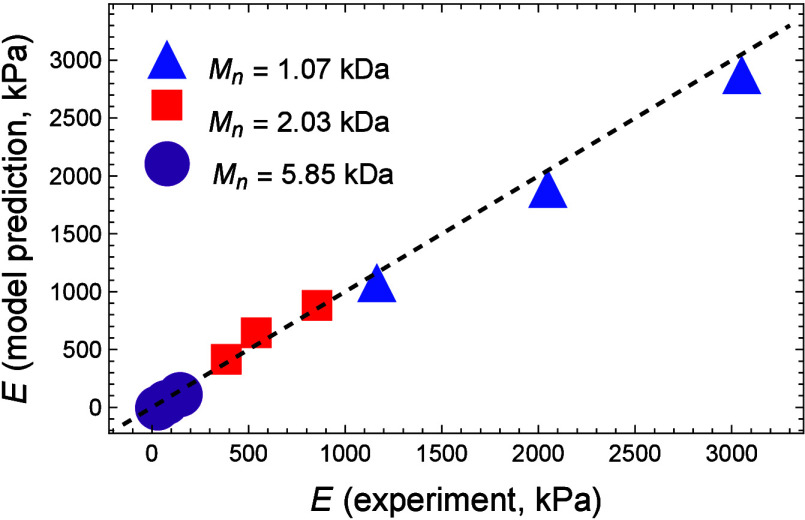
Predicted Young’s moduli (eq 28) as a function of the experimentally measured stiffness *E* for the three molecular weights *M*_*n*_ = 1.07 kDa (triangular markers), *M*_*n*_ = 2.03 kDa (square markers), and *M*_*n*_ = 5.85 kDa (circular markers). The dashed line has a slope of 1 to enable comparison between the experimentally measured and the predicted stiffness. The experimental variations are smaller than the marker size.

A comparison between the Young’s moduli obtained from the experimental data and the proposed model is depicted in Figure 8. Here, the triangle, the square, and the circle marks denote the experimental data and the model prediction (eq 28) for *M*_*n*_ = 1.07 kDa, *M*_*n*_ = 2.03 kDa, and *M*_*n*_ = 5.85 kDa, respectively, and the dashed line has a slope of 1 to enable comparison. Specifically, the distance of the points from the dashed line denotes the error in the model predictions. We find that the model is capable of capturing the stiffness with an error of <10% across a broad range of PEGDA molecular weights. It is emphasized that the stiffness in high molecular weights stems from the entropic contribution of the PEG chains, and therefore the first term in eq 28 is dominant. In lower molecular weights, the PEG chains are short and the PA rods are long, and therefore the stiffness is determined by the PA rods. Specifically, the second term in eq 28 is dominant. As previously described, the interaction term is negligible in the chosen hydrogels.

## 4 Programming the Response of PEGDA Networks

One important advantage of our microscopically motivated model is the ability to investigate the influence of microstructure on the mechanics of the hydrogel network. With the aim of tuning the properties and the response of PEGDA networks, in this section we explore the influence of different microstructural quantities. As above, we set *T* = 300 K, α = *N*_*PA*_, *l*_*A*_ = 0.25 nm, *l*_*p*_ = 17.5 nm, and *l* = 0.3 nm. The influences of the contour length of the PEG chain and the PA rods are studied.

**Figure 9:**
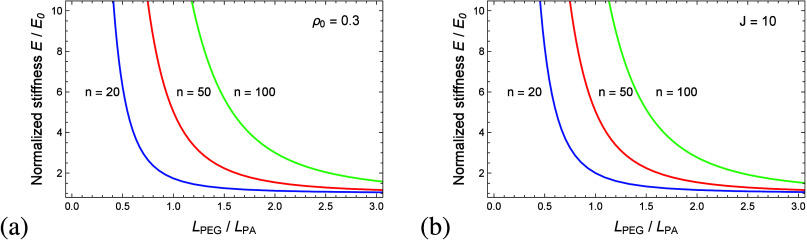
Normalized Young’s modulus *E*/*E*_0_ as a function of the ratio *L*_*PEG*_/*L*_*PA*_ for three representative values of *n* with (a) a constant initial swollen ratio  and (b) a constant water content, defined by the volumetric deformation *J*.

First, we investigate the changes in stiffness as we modify the ratio between the contour length of a PEG chain and that of a PA rod. Figures 9a and 9b plot the normalized Young’s modulus *E*/*E*_0_, where *E*_0_ = 3*N*_*PA*_*k*_*b*_*T*/*J*^1/3^ is the limiting stiffness in the case of *L*_*PEG*_/*L*_*PA*_ → *∞*, as a function of the ratio *L*_*PEG*_/*L*_*PA*_ for three representative values of *n* with a constant initial swollen ratio  and a constant water content, defined by the volumetric deformation *J*, respectively. In the case of a constant ρ_*s*_, the volumetric ratio *J* depends on the contour length of the PEG chains. Maintaining a constant volumetric deformation *J* refers to a network in which the initial end-to-end distance of the chains depends on the number of repeat units in the PEG chain, thereby tuning the initial elasticity of the network.

As expected, in the limit *L*_*PEG*_/*L*_*PA*_ → *∞*, corresponding to PEGDA hydrogels with PEG chains that are significantly longer than the PA rods, the limit *E* = *E*_0_ is recovered. As this ratio decreases, the stiffness of the material increases due to the stiffness of the PA rods and the PA–PA interactions.

We also find that for a given ratio *L*_*PEG*_/*L*_*PA*_, increasing the contour length of the PEG chains yields stiffer PEGDA networks. This trend can be explained as follows: to maintain a constant ratio between the contour length of the PEG chains and the PA rods, an increase in *n* requires an increase in *n*_*PA*_. As a result, such networks comprise longer PA rods that increase the stiffness. Interestingly, under a constant ratio ρ_0_ the volumetric deformation, and consequently the water content, in networks with longer PEG chains is higher. This leads to a slight softening, but the influence of the stiff PA rods dominates. Considering a constant volumetric deformation *J* (and a fixed water content), longer PEG chains experience less stretch due to swelling.^37^ Once again, such response leads to a slight softening, but the influence of the stiff PA rods is dominant. Therefore, an overall stiffening is observed.

**Figure 10:**
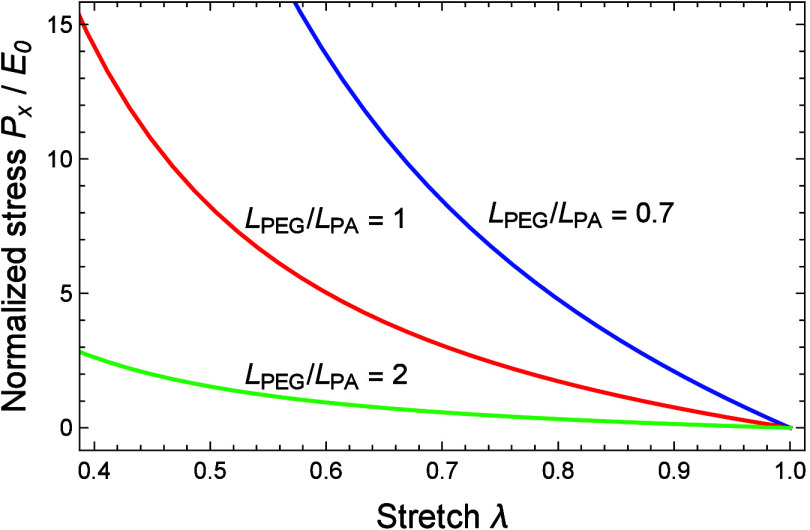
Normalized nominal stress *P*_*x*_/*E*_0_ as a function of the stretch λ for three representative ratios *L*_*PEG*_/*L*_*PA*_.

As previously discussed, the constitutive response to uniaxial compression is governed by the microstructural topology of the PEGDA network and the ratio *L*_*PEG*_/*L*_*PA*_. Figure 10 plots the normalized stress *P*_*x*_/*E*_0_ as a function of the stretch λ for three representative ratios *L*_*PEG*_/*L*_*PA*_ = 0.7, 1, and 2. As expected, decreasing the ratio *L*_*PEG*_/*L*_*PA*_ results in stiffer PEGDA hydrogel networks. As previously mentioned, the response of networks characterized by *L*_*PEG*_/*L*_*PA*_ = 2 is mostly governed by the entropic deformation of the PEG chains while the constitutive behavior of hydrogels with *L*_*PEG*_/*L*_*PA*_ = 0.7 is governed by PA–PA interactions.

## 5 Discussion and Conclusions

In this work, we propose a microstructurally motivated model to explain the origin of the properties and response of PEGDA hydrogels. The unique structure of these gels, which comprise stiff PA rods that interconnect entropic PEG chains, leads to an interesting overall mechanical behavior that is governed by three main mechanisms: (1) the entropic response of the PEG chains, (2) the deformation of the PA rods, and (3) the PA–PA interactions. When subjected to an external loading, the PEG chains elongate or shorten entropically and apply forces on the PA rods, which in turn experience a slight bending. By considering the PEG chains as freely jointed, expressions for the energy associated with these two phenomena is derived. The PA–PA interactions are also crucial to the overall response. Specifically, the deformation of PEGDA networks with short PEG chains and long PA rods is dominated by the mechanical restrictions imposed by the rods. Such restrictions include limited rotational freedom which follows steric interactions due to the anisotropic nature of the PA rods.

The framework results in a model that can be implemented numerically to capture the mechanical response and the microstructural evolution of the network during loading. In addition, closed-form approximations for the Young’s modulus and the stress that develop in networks with long chains and relatively short PA rods are developed.

To validate the model, we fabricated nine experimental hydrogel samples with different molecular weights and concentrations and subjected them to uniaxial compression. The model is found to be in agreement with the experimental findings, thus strengthening its merit. The closed-form solutions provide excellent accuracy for small to moderate deformations with a maximum error of <10% for large compressive in PEGDA networks with low molecular weight.

Our findings show that in hydrogels networks prepared with higher molecular weight PEGDA, the PEG chains are long while the PA rods are short, and accordingly the stiffness and the overall constitutive response is governed by the entropic stretching of the PEG chains. Networks with lower molecular weight PEGDA are characterized by long PA rods and relatively short PEG chains, resulting in a stiffer hydrogel with a constitutive behavior that is governed by the PA rods and the PA–PA interactions.

With the aim of providing a tool for the design of PEGDA networks, we also studied the influence of the ratio *L*_*PEG*_/*L*_*PA*_ between the contour length of the PEG chain and the PA rod on the stiffness and the overall behavior. We show that in the range of *L*_*PEG*_/*L*_*PA*_ ≫ 1, the hydrogel is relatively soft with a response that is mostly entropic. Networks with PEG chains that are shorter than the PA rods are typically stiffer and less extensible due to the PA–PA interactions and the stiffness of the PA rods.

In conclusion, this work sheds light on the underlying structural mechanisms that govern the response of PEGDA hydrogels. Specifically, the findings from this work can be used to program the properties and the response of these gels by appropriate microstructural modifications, including the length of the PEG chains and the PA rods, which are controlled by the molecular weight and the concentration of PEGDA during fabrication. Control over the behavior of PEGDA is crucial for many applications, including tissue engineering, drug delivery, traction force microscopy, and microfluidics. Accordingly, these insights are expected to enhance and even optimize the performance of these materials.

## Supporting Information Available

Experimental video (AVI)
Derivation of the energy-density of PA rods, derivation of a closed form solution for the stress associated with PA rods, experimental parameters, stress components in the PEGDA network, and comparison of the proposed model to solutions from rubber elasticity (PDF)

## Supplementary Material

ma3c02635_si_001.avi
