# Supplementary material for: Understanding the Response of Poly(ethylene glycol) diacrylate (PEGDA) Hydrogel Networks: A Statistical Mechanics-Based Framework
Source: Macromolecules. 2024 Jul 17;57(15):7074–86. doi: 10.1021/acs.macromol.3c02635 (PMC11325651; doi:10.1021/acs.macromol.3c02635)
Supplement: Supplementary file 2 — ma3c02635_si_002.pdf [file ma3c02635_si_002.pdf]

Supplemental information
Understanding the response of Poly(ethylene glycol) diacrylate (PEGDA) hydrogel networks: a statistical mechanics-based framework

Michal Levin[a], Yongkui Tang[b,c], Claus D. Eisenbach[c,d],

Megan T. Valentine[b,c], and Noy Cohen[a,*]

[a]Department of Materials Science and Engineering, Technion - Israel Institute of Technology, Haifa 3200003, Israel

[b]Department of Mechanical Engineering, University of California, Santa Barbara, CA 93106, USA

[c]Materials Research Laboratory, University of California, Santa Barbara, CA 93106, USA

[d]Institut for Polymerchemie, University of Stuttgart, Stuttgart D-70569, Germany

*e-mail address: noyco@technion.ac.il

Contents

1 Derivation of the energy-density of PA rods 3

2 Derivation of a closed form solution for the stress associated with PA rods 6

3 Experimental video - parameters 8

4 Stress components in the PEGDA network 9

5 Comparison of proposed model to solutions from rubber elasticity 11

S1 Derivation of the energy-density of PA rods

In the following, we derive an expression for the energy-density of a PA rod in a PEGDA hydrogel network that is subjected to the entropic forces from n_{PA} PEG chains. We begin by considering a PA rod aligned along a direction $\hat{\mathbf{N}}^{(\alpha)}_{PA}$ in a reference configuration that is connected to n_{PA} PEG chains. It is emphasized that the distribution of the PEG chains differs on all PA rods aligned along the $\hat{\mathbf{N}}^{(\alpha)}_{PA}$-direction. We assume that in all PA rods, the PEG chains apply forces that are perpendicular to the PA axis and are equally spaced with a C-C distance $l_A = 0.25\,\mathrm{nm}$.

To describe the configuration of forces on a PA rod, we define an orthogonal local coordinate system $\left\{\hat{\mathbf{N}}^{(\alpha)}_{PA}, \hat{\mathbf{Y}}^{(\alpha)}_{PA}, \hat{\mathbf{Z}}^{(\alpha)}_{PA}\right\}$ (see Fig. S1a). Next, we examine the K-th PA rod that is aligned along the direction $\hat{\mathbf{N}}^{(\alpha)}_{PA}$. The end-to-end vector of a PEG chain connected to the k-th acrylate unit at a distance $k\, l_A$ from an end of the PA rod points along the direction

$$\hat{\mathbf{R}}^{(\alpha,K,k)} = \cos\left(\varphi^{(\alpha,K,k)}\right) \hat{\mathbf{Y}}^{(\alpha)}_{PA} + \sin\left(\varphi^{(\alpha,K,k)}\right) \hat{\mathbf{Z}}^{(\alpha)}_{PA}, \tag{1}$$

where $0 \leq \varphi^{(\alpha,K,k)} < 2\pi$ is the angle between the end-to-end distance $\hat{\mathbf{R}}^{(\alpha,K,k)}$ and $\hat{\mathbf{Y}}^{(\alpha)}_{PA}$. Note that $\hat{\mathbf{R}}^{(\alpha,K,k)} \perp \hat{\mathbf{N}}^{(\alpha)}_{PA}$. Since the PEG chains are randomly distributed around the PA rod, the angle $\varphi^{(\alpha,K,k)}$ is randomly oriented and uniformly distributed along the $\hat{\mathbf{Y}}^{(\alpha)}_{PA} - \hat{\mathbf{Z}}^{(\alpha)}_{PA}$ plane.

The hydrogel is subjected to an external force, which results in the rotation and deformation

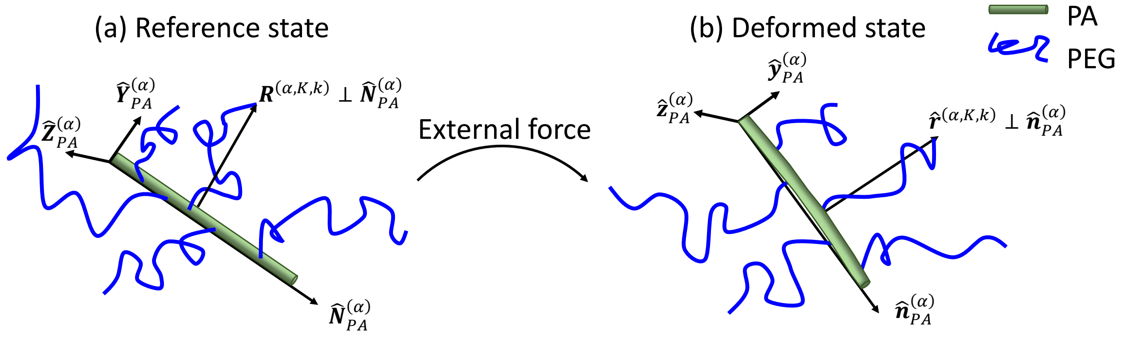

Figure S1: A representative PA rod that interconnects PEG chains in (a) the reference state and (b) the deformed state.

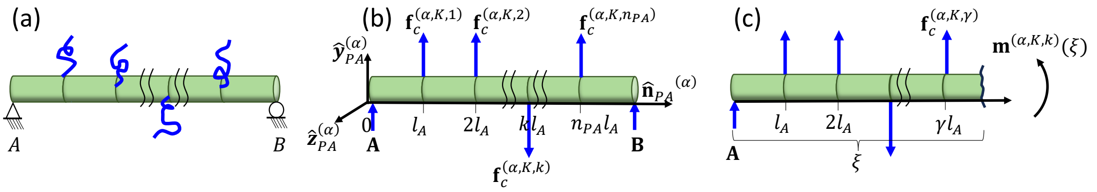

Figure S2: (a) A representative PA rod connected to four PEG chains and supported at points A and B. (b) Free body diagram of the PA rod. Here, $\mathbf{A}$ and $\mathbf{B}$ denote the forces from the supports at points A and B, respectively, and $\mathbf{f}_c^{(\alpha,K,k)}$ is the force exerted by the k-th PEG chain at a distance $k\,l_A$ along the $\hat{\mathbf{n}}_{PA}^{(\alpha)}$ direction. (c) The moment $\mathbf{m}^{(\alpha,K,k)}(\xi)$ at a distance $\gamma l_A \leq \xi < (\gamma+1)\,l_A$ from point A along the PA rod.

of the PEG chains and the PA rods. In the deformed state, we assume that the PEG chains remain perpendicular to the PA rods such that $\hat{\mathbf{r}}^{(\alpha,K,k)} \perp \hat{\mathbf{n}}_{PA}^{(\alpha)}$, where $\hat{\mathbf{n}}_{PA}^{(\alpha)}$ denotes the PA axis direction. As described in the main text, since the PEG chains experience affine deformations, the direction

$$\hat{\mathbf{n}}_{PA}^{(\alpha)} = \frac{\mathbf{F}^{-T}\hat{\mathbf{N}}_{PA}^{(\alpha)}}{\sqrt{\left(\mathbf{F}^{-T}\hat{\mathbf{N}}_{PA}^{(\alpha)}\right)\cdot\left(\mathbf{F}^{-T}\hat{\mathbf{N}}_{PA}^{(\alpha)}\right)}}, \tag{2}$$

where $\mathbf{F}$ is the deformation gradient from the reference to the deformed state.

We define an orthogonal local coordinate system $\left\{\hat{\mathbf{n}}_{PA}^{(\alpha)}, \hat{\mathbf{y}}_{PA}^{(\alpha)}, \hat{\mathbf{z}}_{PA}^{(\alpha)}\right\}$ in the deformed configuration (Fig. S1b) and describe the force applied by a PEG chain at a distance $k\,l_A$ on the K-th chain that is aligned along the $\hat{\mathbf{n}}_{PA}^{(\alpha)}$ direction via

$$\mathbf{f}_c^{(\alpha,K,k)} = \frac{k_b T}{l}\beta\left(\rho^{(\alpha,K,k)}\right)\left(\cos\left(\varphi^{(\alpha,K,k)}\right)\hat{\mathbf{y}}_{PA}^{(\alpha)} + \sin\left(\varphi^{(\alpha,K,k)}\right)\hat{\mathbf{z}}_{PA}^{(\alpha)}\right), \tag{3}$$

To determine the overall energy of the deformed PA rod, we set its two ends on the supports are denoted by A and B, as shown in Fig. S2a. Equilibrium of linear and angular momenta results in the forces exerted by the supports, which are denoted by $\mathbf{A}$ and $\mathbf{B}$ in S2b,

$$\mathbf{A} = -\sum_{k=1}^{n_{PA}}\left(1 - \frac{k}{(n_{PA}+1)}\right)\mathbf{f}_c^{(\alpha,K,k)}; \quad \mathbf{B} = -\sum_{k=1}^{n_{PA}}\frac{d}{(n_{PA}+1)}\mathbf{f}_c^{(\alpha,K,k)}. \tag{4}$$

Next, the internal moment that develops at a distance $\gamma\,l_A \leq \xi < (\gamma+1)\,l_A$ from point A in order to maintain equilibrium, as demonstrated in Fig. S2c, is

$$\mathbf{m}^{(\alpha,K,k)}\left(\xi\right)=\hat{\mathbf{n}}_{PA}^{(\alpha)}\times\left(\sum_{k=1}^{\gamma}\left(-\frac{\xi\,k}{n_{PA}+1}+k\,l_{A}\right)\mathbf{f}_{c}^{(\alpha,K,k)}+\sum_{k=\gamma+1}^{n_{PA}}\left(\xi-\frac{\xi\,k}{n_{PA}+1}\right)\mathbf{f}_{c}^{(\alpha,K,k)}\right). \tag{5}$$

Consequently, the energy-density of a PA rod along the $\hat{\mathbf{n}}_{PA}$-direction can be computed via

$$\psi_{r}^{(\alpha,K)}=\frac{1}{2k_{b}Tl_{p}}\sum_{\gamma=1}^{n_{PA}}\int\limits_{\gamma l_{A}}^{(\gamma+1)l_{A}}\left(\mathbf{m}^{(\alpha,K,k)}\left(\xi\right)\cdot\mathbf{m}^{(\alpha,K,k)}\left(\xi\right)\right)d\xi, \tag{6}$$

where $l_p = EI/k_bT$ is the persistence length of the PA chain and EI is the bending stiffness.

S2 Derivation of a closed form solution for the stress associated with PA rods

In the following we derive an approximation the stress associated with the PA rods in the PEGDA hydrogel network, which is given in Eq. 18 of the main manuscript.

First, we recall the stress associated with the average PA rod that is aligned along the $\hat{\mathbf{N}}_{PA}^{(\alpha)}$ direction in the reference configuration. As shown in Eq. 15, this stress can be written as

$$\boldsymbol{\sigma}_r^{(\alpha)} = \frac{1}{J} N_{PA}^{(\alpha)} \frac{k_b T}{2\, l_p\, l^2} \mathbf{F} \left(\int_0^{2\pi} \boldsymbol{\eta} \frac{1}{2\pi} \mathrm{d}\varphi \right) \mathbf{F}^T, \tag{7}$$

where

$$\boldsymbol{\eta} = \sum_{\gamma=1}^{n_{PA}} \int_{\gamma l_A}^{(\gamma+1) l_A} \left(\sum_{k=1}^{\gamma} \left(-\frac{\xi\, k}{n_{PA}+1} + k\, l_A \right)^2 A\, \hat{\mathbf{R}} \otimes \hat{\mathbf{R}} + \sum_{k=\gamma+1}^{n_{PA}} \left(\xi - \frac{\xi\, k}{n_{PA}+1} \right)^2 A\, \hat{\mathbf{R}} \otimes \hat{\mathbf{R}} \right) \mathrm{d}\xi, \tag{8}$$

and $A = \left(2\beta\, \mathrm{d}\beta / \mathrm{d}r\right) / \left(\rho\, n\right)$, with $\mathrm{d}\beta/\mathrm{d}r = \beta^2 \sinh^2\beta / \left(\sinh^2\beta - \beta^2\right)$. The total overall stress associated with all PA rods in the network is (Eq. 18 in the main manuscript)

$$\boldsymbol{\sigma}_{PA} = N_{PA} \left\langle \boldsymbol{\sigma}_r \right\rangle . \tag{9}$$

To approximate the total stress, we take a second order Taylor series of the quantity A around $\rho = 0$, which yields

$$A \approx \frac{18}{n} + \frac{222}{5n} \rho^2 + o\left(\rho^4\right). \tag{10}$$

Here, the ratio

$$\rho = \frac{1}{\sqrt{n}} \sqrt{\mathbf{F}\hat{\mathbf{R}} \cdot \mathbf{F}\hat{\mathbf{R}}}. \tag{11}$$

Next, we substitute this approximation into Eq. 7 to obtain $\boldsymbol{\sigma}_r^{(\alpha)}$, and determine the total

stress $\boldsymbol{\sigma}_{PA}$ with Eq. 9. This process leads to

$$\boldsymbol{\sigma}_{PA} \approx \frac{N_{PA} k_b T}{2\, l_p\, l^2 n} \eta \left(18\mathbf{F} \left\langle \hat{\mathbf{R}} \otimes \hat{\mathbf{R}} \right\rangle \mathbf{F}^T + \frac{222}{5n} \mathbf{F} \left\langle \left(\mathbf{F}\hat{\mathbf{R}} \cdot \mathbf{F}\hat{\mathbf{R}} \right) \hat{\mathbf{R}} \otimes \hat{\mathbf{R}} \right\rangle \mathbf{F}^T \right), \tag{12}$$

where

$$\eta = \sum_{\gamma=1}^{n_{PA}} \int_{\gamma l_A}^{(\gamma+1) l_A} \left(\sum_{k=1}^{\gamma} \left(-\frac{\xi\, k}{n_{PA}+1} + k\, l_A \right)^2 + \sum_{k=\gamma+1}^{n_{PA}} \left(\xi - \frac{\xi\, k}{n_{PA}+1} \right)^2 \right) \mathrm{d}\xi. \tag{13}$$

Since the chains are initially randomly oriented and uniformly distributed, the first average $\left\langle \hat{\mathbf{R}} \otimes \hat{\mathbf{R}} \right\rangle = 1/3\mathbf{I}$. Accordingly, $\mathbf{F} \left\langle \hat{\mathbf{R}} \otimes \hat{\mathbf{R}} \right\rangle \mathbf{F}^T = 1/3\, \mathbf{F}\mathbf{F}^T$. To determine the second average, it is convenient to rewrite it using index notations,

$$\begin{aligned} \left(\mathbf{F} \left\langle \left(\mathbf{F}\hat{\mathbf{R}} \cdot \mathbf{F}\hat{\mathbf{R}} \right) \hat{\mathbf{R}} \otimes \hat{\mathbf{R}} \right\rangle \mathbf{F}^T \right)_{\alpha\beta} &= F_{\alpha\gamma} F^T_{\delta\beta} F_{ij} F_{ik} \left\langle \hat{R}_j \hat{R}_k \hat{R}_\gamma \hat{R}_\delta \right\rangle \\ &= \frac{1}{15} \left(I_1 F_{\alpha\gamma} F^T_{\gamma\beta} + 2 F_{\alpha\gamma} F^T_{\gamma i} F_{i\delta} F^T_{\delta\beta} \right), \end{aligned} \tag{14}$$

where $I_1 = F_{ik} F^T_{ki} = \mathrm{Tr}\left(\mathbf{F}\mathbf{F}^T \right)$ is the first invariant and the relation

$$\left\langle \hat{R}_j \hat{R}_k \hat{R}_\gamma \hat{R}_\delta \right\rangle = 1/15 \left(\delta_{jk}\delta_{\gamma\delta} + \delta_{j\gamma}\delta_{k\delta} + \delta_{j\delta}\delta_{k\gamma} \right)$$

is employed. Here, δ_{ij} denotes the Kronecker delta. Accordingly,

$$\mathbf{F} \left\langle \left(\mathbf{F}\hat{\mathbf{R}} \cdot \mathbf{F}\hat{\mathbf{R}} \right) \hat{\mathbf{R}} \otimes \hat{\mathbf{R}} \right\rangle \mathbf{F}^T = \frac{1}{15} \left(I_1 \mathbf{F}\mathbf{F}^T + 2\mathbf{F}\mathbf{F}^T\mathbf{F}\mathbf{F}^T \right). \tag{15}$$

Substituting the averages into Eq. 12 and rearranging the terms yields

$$\boldsymbol{\sigma}_{PA} \approx \frac{N_{PA} k_b T}{l_p l^2 n} \eta \left(\left(3 + \frac{37}{25n} I_1 \right) \mathbf{F}\mathbf{F}^T + \frac{74}{25n} \mathbf{F}\mathbf{F}^T\mathbf{F}\mathbf{F}^T \right), \tag{16}$$

which is Eq. 23 in the main manuscript.

S3 Experimental video - parameters

The supplemental video SV1 shows a side view of a uniaxial compression test on a cylindrical PEGDA hydrogel sample with an initial radius of $6\,\mathrm{mm}$, molecular weight $M_n = 2.03\,\mathrm{kDa}$, and concentration $c = 18.4\,\%\mathrm{wt/wt}$. The polymer content is ~ 0.132, corresponding to a ratio of $J = 7.6$ between the swollen and the dry states.

S4 Stress components in the PEGDA network

To emphasize the influence of the different stress components, we plot the contribution of the stress components P_{PEG}, P_{PA}, and P_{int} due to the deformation of the PEG network, the bending of the PA rods, and the PA-PA interactions, respectively, as a function of the stretch λ for hydrogel networks in Figs. S3a-c for three representative PEGDA networks. In the case of $M_n = 2.03\,\mathrm{kDa}$ and $c = 15.8\,\%wt/wt$, the PEG chains are shorter than the PA rods. Accordingly, the stress component P_{PA} associated with the bending of the PA rods governs the response of the network (see Fig. S3a). In networks characterized by $M_n = 5.85\,\mathrm{kDa}$ and the concentrations $c = 8.0\,\%wt/wt$ and $c = 15.9\,\%wt/wt$, as shown in Fig. S3b and S3c, the chains are significantly longer than the PA rods. However, the water content in the two PEGDA networks is significantly different. In networks with $c = 8.0\,\%wt/wt$, $J = 35.7$ and, in order to accommodate the presence of the water molecules, chains experience a larger stretch and therefore stiffen significantly and govern the response. In networks with $c = 15.9\,\%wt/wt$, the volumetric deformation is $J = 16.9$. Interestingly, in this hydrogel the stress contributions P_{PEG} and P_{PA} are similar. In all cases the interaction energy is negligible. It is emphasized that such an energy is expected to be more dominant as the ratio $L_{PEG}/L_{PA} \to 0$.

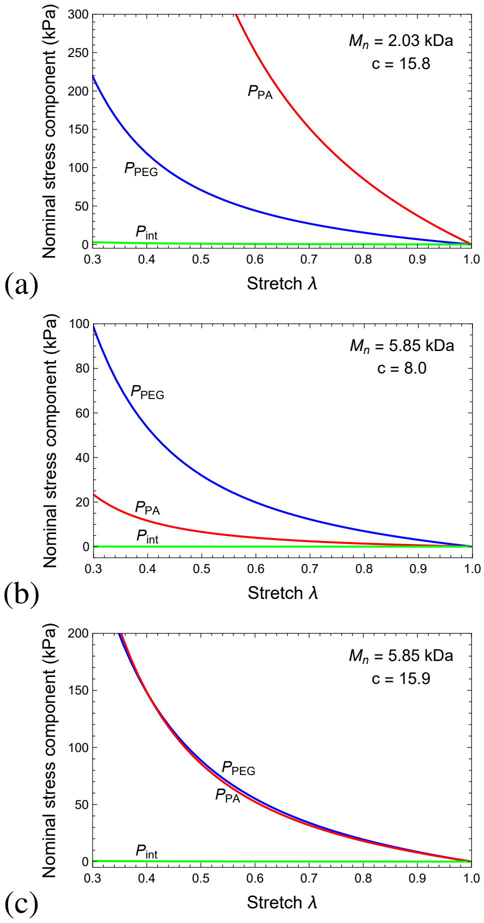

Figure S3: The stress contributions P_{PEG}, P_{PA}, and P_{int} due to the deformation of the PEG network, the bending of the PA rods, and the PA-PA interactions as a function of the stretch λ for hydrogel networks with (a) $M_n = 2.03\,\mathrm{kDa}$ and $c = 15.8\,\%wt/wt$, (b) $M_n = 5.85\,\mathrm{kDa}$ and $c = 8.0\,\%wt/wt$, and (c) $M_n = 5.85\,\mathrm{kDa}$ and $c = 15.9\,\%wt/wt$.

S5 Comparison of proposed model to solutions from rubber elasticity

In the following, we compare the model predictions to the classical models that assume point-like junctions. Specifically, we neglect the terms associated with the bending of the PA rods and the interaction energy and compute the true stress $\boldsymbol{\sigma}_{PEG}$, as written in Eq. 8, and the nominal stress $\mathbf{P}_{PEG}$.

Figs. S4a-c plot the nominal stress P_x as a function of the stretch λ for PEGDA networks with different concentrations and molecular weights $M_n = 1.07\,\mathrm{kDa}$, $M_n = 2.03\,\mathrm{kDa}$, and $M_n = 5.85\,\mathrm{kDa}$, respectively. The continuous and the dashed curves denote the predictions of the full model and the solutions from rubber elasticity for a network with point-like junctions, respectively.

In the case of low molecular weights, the PA rods are longer than the PEG chains and the response is dominated by the bending of the stiff backbone. Therefore, as can be seen in Figs. S4a and S4b, the classical solutions predict a significantly softer mechanical response and are not capable of capturing the behavior of the PEGDA networks. A similar trend is seen in networks that experience moderate swelling with a higher molecular weight (see red and green curves in Fig. S4c for $M_n = 5.85\,\mathrm{kDa}$). In such cases, the stiffening of the chains due to the stretching in the presence of water molecules is not sufficient to govern the response. However, the contribution of the PA rods can be neglected in networks with PEG chains that are significantly longer than the PA rods, highly swollen, and stretched. This is shown by the blue curve in Fig. S4c, where the influence of the PA rods can be neglected to result in a small error.

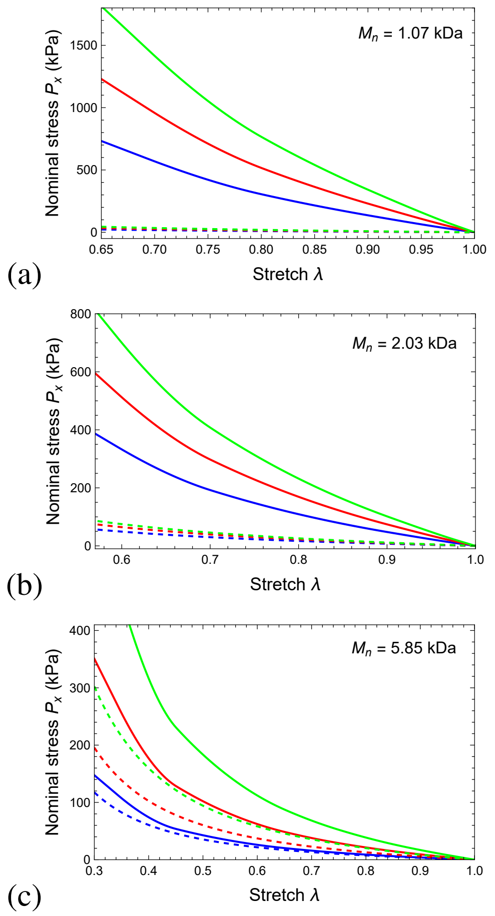

Figure S4: Comparison of the model to the classical solution from rubber elasticity for (a) $M_n = 1.07\,\mathrm{kDa}$, (b) $M_n = 2.03\,\mathrm{kDa}$, and (c) $M_n = 5.85\,\mathrm{kDa}$. The continuous and the dashed curves denote the predictions of the full model and the solutions from rubber elasticity for a network with point-like junctions, respectively.
